# Editorial: Mini symposium on obstetric imaging

**DOI:** 10.4103/0971-3026.43851

**Published:** 2008-11

**Authors:** Milind S Gune, BS Rama Murthy

**Affiliations:** Gune Medical Center, Ambarnath (Dist-Thane), Maharashtra, India; 1 Srinivasa Ultrasound Scanning Center, Bangalore, India

It is indeed a great privilege for us to be the joint editors of this special issue dedicated to obstetric USG and imaging. In the mid and late eighties, when USG made its debut in our country, the machines had linear and mechanical sector transducers. Only rarely did a department have an endocavitary transducer (and an extremely bulky one at that). Some departments still had static USG machines. From those early days of USG in India, we have journeyed to digital machines with phased sector and convex arrays, color Doppler machines, and machines with volume imaging capabilities. Today, the high-end machines generate spectacular images. The process of technological innovation is never ending and just when we think that the ultimate has been reached, something better comes along. These advances have exposed the fetus to scrutiny like never before. This has exponentially increased the onus on the radiologist. In addition to imaging skills, the practicing radiologist should now possess a sound knowledge of genetics, fetal pathophysiology, and obstetric management. Legal, ethical, and regulatory issues are other dimensions that now form an integral part of obstetric imaging practice. It is the radiologist who becomes the sheet anchor in coordinating multispecialty management of fetal disorders. He is the specialist whom the prospective parents and the referring clinician turn to whenever there is a doubt. Indeed, it is due to these unique responsibilities that the radiologist evolves into a fetal medicine specialist. 

Obstetric imaging practice is governed by the Preconception and Prenatal Diagnostic Techniques Act 1994. Radiologists have to fall in line and comply with the requirements of the Act. None of our community should misuse USG. Let us not abet crime against the innocent unborn girl child. 

Today the field of obstetric imaging is being encroached upon by non-radiologist practitioners who are often ill trained. The only way to stem this encroachment is to adhere to the highest standards of practice. The highly demanding nature of obstetric imaging should act as a stimulus rather than as a deterrent to the young radiologist. It is pure fascination and challenge that should inspire our young consultants to specialize in obstetric imaging and fetal medicine. If obstetric USG as a subspecialty is to attract the best of our young radiology talent, efforts must be undertaken to provide quality training.

In terms of quality and comprehensibility, the images today are intelligible to the average pregnant subject and her husband; this not only applies to 3D surface rendering but also to 2D grayscale images. The fetal cranium, heart, limbs, and spine are easily recognized by the prospective parents. The request of a couple to have a sneak preview of their unborn child has to be respected by the operator. This will reinforce the verbal and written communication regarding the normalcy of the fetus. Such a demonstration is best limited to 2D images on the monitor during or after the examination. Occasionally a lady may want to listen to her baby's heartbeat. Usage of spectral Doppler for this purpose must be discouraged. The USG energy deposited into the region being scanned is the greatest during spectral Doppler examination.

There are more and more requests by couples to view their unborn babies by 3D and 4D USG. Such services are now being offered for pecuniary gain. Indeed, this has resulted in the proliferation of ‘3D/4D USG centers.’ In this context we must ask ourselves whether we have become medical imaging specialists merely to cater to social demands or whether we are just social photographers of the fetus? We strongly feel that this practice is to be abhorred; 3D or 4D USG should only be done for a medical indication. The practice of issuing images and clips on compact discs is fast picking up. The pros and cons of this practice need to be seriously debated. Standardization of obstetric USG (i.e., creation of an Indian obstetrical USG standards or guidelines) is another area where a consensus within the radiology community should be reached. It is well recognized that many anomalies cannot be detected using USG because there are so many maternal-, fetal-, technical-, and operator-dependent factors. A certain level of standardization will go a long way in minimizing preventable errors and increasing the pickup rate.

We have derived enormous satisfaction in putting together the articles for this mini symposium. Our heartfelt thanks go out to all the contributors, international and national, for their time, effort, and expertise. We hope that you enjoy and benefit from this endeavor.

